# Molecular Characterization and Patient Outcome of Melanoma Nodal Metastases and an Unknown Primary Site

**DOI:** 10.1245/s10434-014-3799-y

**Published:** 2014-05-28

**Authors:** Aleksandra Gos, Monika Jurkowska, Alexander van Akkooi, Caroline Robert, Hanna Kosela-Paterczyk, Senada Koljenović, Nyam Kamsukom, Wanda Michej, Arkadiusz Jeziorski, Piotr Pluta, Cornelis Verhoef, Janusz A. Siedlecki, Alexander M. M. Eggermont, Piotr Rutkowski

**Affiliations:** 1 Department of Molecular and Translational Oncology, Maria Sklodowska-Curie Memorial Cancer Center and Institute of Oncology, Warsaw, Poland; 2 Department of Soft Tissue/Bone Sarcoma and Melanoma, Maria Sklodowska-Curie Memorial Cancer Center and Institute of Oncology, Warsaw, Poland; 3 Department of Pathology, Maria Sklodowska-Curie Memorial Cancer Center and Institute of Oncology, Warsaw, Poland; 4 Institute of Rheumatology, Warsaw, Poland; 5 Department of Surgical Oncology, Erasmus MC Cancer Institute, Rotterdam, The Netherlands; 6 Department of Pathology, Erasmus University Medical Centre, Rotterdam, The Netherlands; 7 Cancer Institute Gustave Roussy, Villejuif/Paris-Sud, France; 8 Medical University of Lodz, Lodz, Poland

## Abstract

**Background:**

Melanoma of unknown primary site (MUP) is not a completely understood entity with nodal metastases as the most common first clinical manifestation. The aim of this multicentric study was to assess frequency and type of oncogenic *BRAF*/*NRAS*/*KIT* mutations in MUP with clinically detected nodal metastases in relation to clinicopathologic features and outcome.

**Materials and Methods:**

We analyzed series of 103 MUP patients (period: 1992–2010) after therapeutic lymphadenectomy (LND): 40 axillary, 47 groin, 16 cervical, none treated with BRAF inhibitors. We performed molecular characterization of *BRAF*/*NRAS*/*KIT* mutational status in nodal metastases using direct sequencing of respective coding sequences. Median follow-up time was 53 months.

**Results:**

*BRAF* mutations were detected in 55 cases (53 %) (51 V600E, 93 %; 4 others, 7 %), and mutually exclusive *NRAS* mutations were found in 14 cases (14 %) (7 p.Q61R, 4 p.Q61K, 2 p.Q61H, 1 p.Q13R). We have not detected any mutations in *KIT.* The 5-year overall survival (OS) was 34 %; median was 24 months. We have not found significant correlation between mutational status (*BRAF*/*NRAS*) and OS; however, for *BRAF* or *NRAS* mutated melanomas we observed significantly shorter disease-free survival (DFS) when compared with wild-type melanoma patients (*p* = .04; 5-year DFS, 18 vs 19 vs 31 %, respectively). The most important factor influencing OS was number of metastatic lymph nodes >1 (*p* = .03).

**Conclusions:**

Our large study on molecular characterization of MUP with nodal metastases showed that MUPs had molecular features similar to sporadic non-chronic-sun-damaged melanomas. *BRAF*/*NRAS* mutational status had negative impact on DFS in this group of patients. These observations might have potential implication for molecular-targeted therapy in MUPs.

Metastatic involvement of lymph nodes is the most common clinical manifestation of melanoma of unknown primary site (MUP) and accounts for ~3.2 % of all melanoma cases, ranging from 1 to 15 % of all melanomas with clinically detectable synchronous lymph nodes involvement.^1^–^7^ Currently, according to the American Joint Committee on Cancer (AJCC) guidelines, presentation of initial metastases in the lymph nodes should be presumed to be regional and staged accordingly (stage III instead of stage IV), if no other site of metastases is discovered during screening process.^8^ We hypothesize, the pathogenesis and molecular characteristics of MUP should be similar to melanomas with nodal metastases from known primary site.

It is now becoming clear that melanoma is not a homogeneous disease, but rather a group of neoplasms caused by different genetic changes and driven by different mechanisms. Patterns of known genetic changes differ significantly based on location of primary lesion and clinical melanoma subtype. Products of genes most commonly alternated in melanoma arising from the skin without chronic sun damage (NCSD, non-chronic sun damaged) are clustered in mitogen activated protein kinase (MAPK) pathway.^9^–^11^ In a majority of cases, the hyperactivation of MAPK pathway is caused by acquisition of oncogenic mutation in *BRAF* or *NRAS* genes. *BRAF* mutations are the most frequent changes in melanoma, and they comprise 40–70 % of cases depending on melanoma type.^12^–^14^ More than 50 distinct mutations of *BRAF* gene were reported; ~90 % of them are due to a single nucleotide substitution T1799A at codon position 600 in exon 15 (p.V600E) leading to 500-fold increase in the protein activity. The second most frequent mutation is p.V600K, and it is less powerful as kinase activity increases. The important role of *BRAF* alternations in melanoma development is proven; however, the mutation itself is not sufficient for malignant transformation, and *BRAF* mutations also occur with high frequency in benign nevi.^15^

The frequency of *NRAS* mutation in melanoma of cutaneous origin varies between 15 and 30 %.^16^ The majority of changes in this gene affect codon 61 (exon 2) as well as, to a lesser extent, codons 12 and 13 (exon 1). Similarly to *BRAF*, the *NRAS* mutations role in tumorigenesis is proven; however again, alone they are not sufficient to cause malignant transformation. *BRAF* and *NRAS* mutations are mutually exclusive, which suggests functional redundancy in primary tumors. *KIT* alterations are rare and found mainly in melanomas on chronically sun-damaged skin, in acral-lentiginous or mucosal type.^9^

The prognostic role of alternations in *BRAF* and *NRAS* genes is not yet determined. Some reports imply association of *BRAF*/*NRAS* mutations and poorer prognosis in the metastatic setting. However, differences in disease-free survival (DFS) and overall survival (OS) according to *BRAF*/*NRAS* mutational status are not seen, when calculated from primary tumor diagnosis.^11^,^17^,^18^ Also data on survival in stage III melanoma patients according to *NRAS* mutations are not unanimous.^18^,^19^ Mutations in both genes are validated targets for molecular-targeted therapies in melanoma (BRAF inhibitors vemurafenib and dabrafenib for *BRAF* mutants and MEK inhibitor trametinib for genotype containing any of the 2 genes altered).^20^–^22^ However, the molecular background of MUP, its linkage to clinical data, and differences from melanoma of known primary site are not fully understood, although they have been explored in recent series.^23^,^24^

In the current study we analyzed frequency and type of oncogenic mutations in known oncogenes (*BRAF*, *NRAS*, and *KIT*) involved in melanoma development in large contemporary series of MUP patients with long follow-up, and we correlated these outputs with disease clinical features and outcome. It may provide insight into molecular pathogenesis and characterization of this melanoma subtype.

## Materials and Methods

### Patients Characteristics

Patients were considered eligible for the study if they had diagnosed clinical (palpable) nodal metastases of MUP, available tumor tissue, and underwent radical lymph node dissection (LND) at 1 of the centers participating in the study [Cancer Centre and Institute of Oncology, Warsaw, Poland (CCIO); Cancer Institute Gustave Roussy, Villejuif, France (IGR); Erasmus MC Cancer Institute, Rotterdam, Netherlands (Erasmus MC), and Medical University of Lodz, Poland (MU)].

The group of patients with MUP and lymph node metastases was defined as: metastases to the lymph nodes as first site of metastases, confirmed clinically, cytologically/histologically, and immunohistochemically; the absence of previous cutaneous tumors or melanomas of unusual primary sites; no prior excisions or destruction of skin lesions without a pathologic examination; and no other detectable metastases at diagnosis, after a detailed checkup that included computed tomography imaging (neck, chest, and abdomen).

Radical LNDs were performed between January 1992 and November 2010.

For the final examination, 103 formalin-fixed, paraffin-embedded tumor tissues from MUP lymph node metastases were selected, after pathological confirmation (40 from CCIO, 7 from IGR, 52 from Erasmus MC, and 4 from MU). There was access to complete clinical data including date of LDN, date of disease relapse, last follow-up, or death for all patients.

Patient characteristics are summarized in Table 1. The study was approved by the local Bio-Ethics Committee according to Good Clinical Practice Guidelines.

**Table 1:** Patient characteristics and comparison between *BRAF*-mutant and *BRAF* wild-type melanomas in stage III melanomas with unknown primary site

	*N* (%) 103 (100 %)	*BRAF* mutants	*BRAF* wild type *N* (%) 48	*p* value *BRAF* + versus −
		*N* (%) 55		
Age, median (years)	54.5	51.5	56.5	ns (*p* = .08)
Age groups (years)				ns
0–40	21 (17.6)	13 (23.7)	9 (18.8)	
>40–60	45 (47.2)	27 (49.0)	18 (37.5)	
60	36 (35.2)	15 (27.3)	21 (43.7)	
Gender				ns
Female	47 (45.6)	25 (45.5)	22 (45.8)	
Male	56 (54.4)	30 (54.5)	26 (54.2)	
Center				ns
CCIO Warsaw	40 (39)	25 (45)	15 (31)	
Erasmus MS, Rotterdam	52 (50)	25 (45)	27 (56.5)	
IGR, Paris	7 (7)	3 (6)	4 (8.5)	
MU, Lodz	4 (4)	2 (4)	2 (4)	
Lymph nodal basin				ns
Axillary	40 (39)	25 (45)	15 (31)	
Inguinal	47 (46)	23 (42)	24 (50)	
Cervical	16 (15)	7 (13)	9 (19)	
Number of metastatic nodes				ns
1	37 (36)	18 (32)	19 (40)	
2–3	20 (19)	12 (21)	8 (17)	
≥4	46 (45)	19 (34)	27 (58)	
Median	3	3	3	
Extracapsular extension of metastatic node (data not available in 40 cases)				ns
Present	27 (43)	15 (38)	12 (50)	
Absent	36 (57)	24 (62)	12 (50)	

*ns* not significant statistically

The patients had not undergone any other preliminary selection. Only patients who met with all the conditions listed previously were enrolled in the study. All patients were followed carefully with a median follow-up time of 53 months for survivors (range 6–140 months) with standard postoperative follow-up protocol (surveillance recommended every 3 months for the first 2 years, every 4 months in year 3, every 6 months for years 4–5, and annually thereafter). Patients were not treated with any BRAF or MEK inhibitors.

### Mutational Testing

A total of 103 paraffin blocks from lymph nodes metastases were selected (1 per patient), with the sufficient tumor load and best possible material quality, as described previously.^23^ Samples were cut from the whole block surface. Genomic DNA was isolated with the Sherlock AX DNA kit (A&A Biotechnology, Gdynia, Poland) and amplified in standard polymerase chain reaction conditions with in-house designed primers for *BRAF* exons 11, 15, *NRAS* exons 1, 2, and *KIT* exons 9, 11, 13, 17. Products were bidirectly sequenced with the BigDye Terminator Cycle Sequencing Kit and ABI Prism 3100 Genetic Analyzer (Applied Biosystems, Carlsbad, CA, USA). In order to identify mutations, the sequences were aligned to the *BRAF* (NM_004333.4), *NRAS* (NM_002524.4), and *KIT* (NM_000222.2) GenBank references.

### Statistical Analyses

All statistical analyses were performed using R 2.15.1 statistical software (R Core Team (2012); http://www.R-project.org). Contingency tables were analyzed using the Chi square test. The nonparametric Mann–Whitney *U* test was applied for comparisons of 2 groups with non-normal distribution.

For survival analyses, the Kaplan–Meier estimator was used with log-rank tests for bivariate comparisons. OS time for the assessment of prognostic value of clinical, pathological, and molecular parameters was calculated from the date of LND to the date of the most recent follow-up (censored data) or death. DFS time was calculated from the date of therapeutic lymphadenectomy to the date of the most recent follow-up or disease recurrence.

The following clinical, pathological, and molecular parameters were tested as potential factors affecting patient survival: gender, age (≤40 vs 40–60 vs >60 years), LND localization (groin vs axillary vs cervical), number of lymph nodes with metastases (1 vs 2–3 vs ≥4), presence of extracapsular invasion in the involved lymph nodes, *BRAF* status (*BRAF* mutated vs wild type), and *NRAS* status (*NRAS* mutated vs wild type).

The differences were considered statistically significant if the respective *p* values were <.05.

## Results

### Mutational Status and Correlation with Clinicopathological Features

*BRAF* mutations were detected in 55 of 103 cases (53.4 %) of melanoma nodal metastases. Majority of mutations (54 of 55) affected codon 600: 51 were p.V600E (92.7 %), 2 were p.V600 K (3.6 %), and 1 was p.V600_K601delinsE codon deletion (1.8 %). The only mutation outside codon 600 was p.E586K. A total of 48 samples were wild type for *BRAF* exons 11 and 15.

The analysis of *NRA*S gene status revealed 14 mutated cases, 13 mutations affected codon 61 (7 p.Q61R, 4 p.Q61K, 2 p.Q61H) and 1 affected codon 13 (p.Q13R). *NRAS* gene was mutated in 13.7 % of all samples, 29 % of *BRAF*-*WT* samples. Mutations in *BRAF* and *NRAS* were mutually exclusive, and 34 samples harbored neither. No *KIT* mutations were detected in analyzed cases.

All cases with weak (<30 % of *BRAF*/*NRAS* wild-type) mutation sequence peak were resequenced, resulting in complete confirmation of the results.

The presence of *BRAF* mutation had trended to correlate only with younger age of patients, with borderline significance (median, 51.5 vs 56.5 years for *BRAF*+ vs *BRAF*−; *p* = .08) (Table 1). We found no differences in patient characteristics when analyzed according to *NRAS* mutational status. There were no differences in terms of mutation distribution between participating centers.

### Treatment Outcomes

**Fig. 1:**
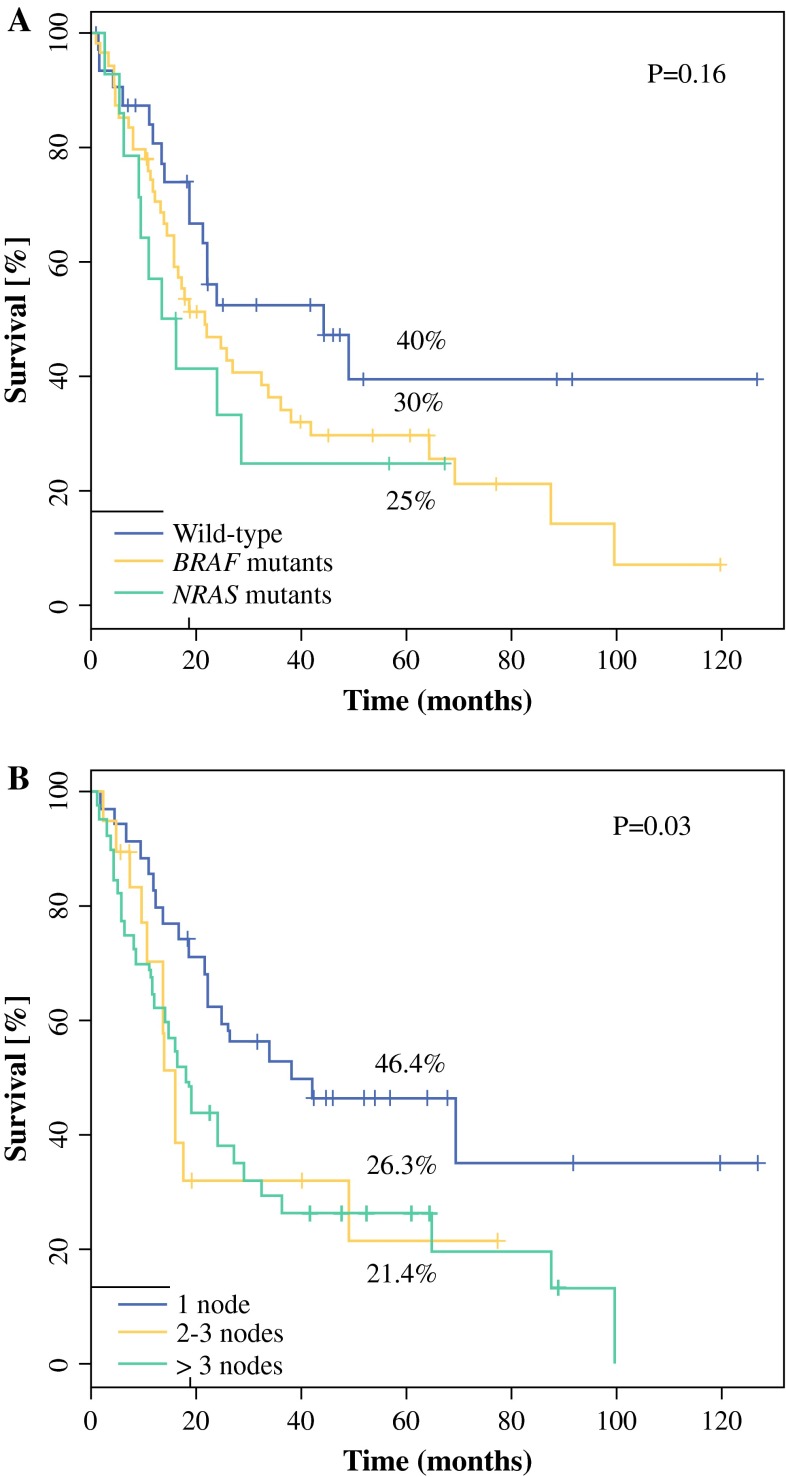
Overall survival according to: **a** *BRAF* and *NRAS* mutational status in melanomas with unknown primary site and nodal involvement and **b** to number of nodes with metastases

The 5-year OS and DFS rates were 34 % [95 % confidence interval (95 % CI) 25–46 %] and 24 % (95 % CI 16–35 %), respectively; median OS and DFS were 24 months (95 % CI 18.2–36.2 months) and 9 months (95 % CI 6–12 months). There were no significant differences in OS and DFS between patients with *BRAF*- or *NRAS*-mutated melanoma and those with no mutation. The 5-year survival rates were 36.0 and 29.8 % for patients with wild-type and mutated *BRAF* genotype (*p* = .27), respectively; 33.9 and 26.0 % for patients with wild-type and mutated *NRAS* genotype (*p* = .31), respectively. Median OS for patients with *BRAF* mutation versus *NRAS* mutation versus wild-type were: 21.9 versus 15.1 versus 43.3 months, respectively. The trend for patients with acknowledged mutations to have worse OS has not reached statistical significance (*p* = .16) (Fig. 1a).

The only negative factor that influenced OS significantly was the number of lymph nodes involved >1 (5-year OS rates, 46.4 vs 21.4 vs 26.3 % for 1 vs 2–3 vs >3 metastatic lymph nodes, respectively; *p* = .03) (Fig. 1b). There was also a trend for poorer survival in patients with extracapsular extension of nodal metastases, but it did not reach statistical significance (5-year OS rates, 47 vs 33.5 % for absence and presence of extracapsular involvement, respectively; *p* = .1).

**Fig. 2:**
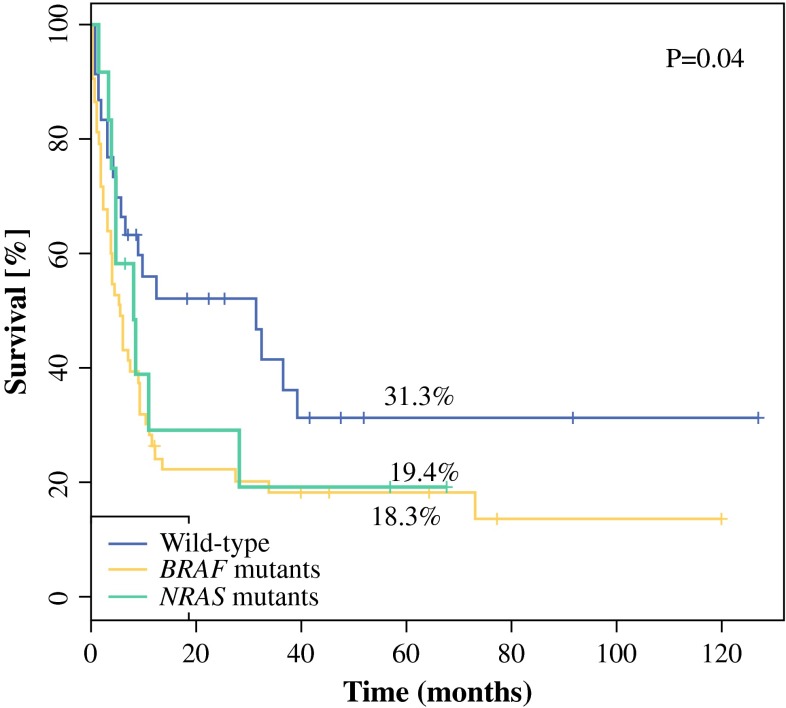
Disease-free survival (DFS) according to *BRAF* and *NRAS* mutational status in melanomas with unknown primary site and nodal involvement

The 5-year DFS rates were 28.5 and 18.3 % for patients without and with *BRAF* mutation, respectively (median DFS, 11.8 vs 5.6 months; *p* = .03), and 31.3, 18.3, and 19.4 % for patients without any mutation and with gene alternations in *BRAF* and *NRAS,* respectively (median, 31.5 vs 5.6 vs 8.3 months; *p* = .04) (Fig. 2). Presence of *BRAF*/*NRAS* mutations was related to significantly shorter DFS.

## Discussion

In this study, we analyzed the large and clinically homogeneous group of lymph node metastases that were the first clinical manifestation of advanced melanoma without known primary origin. We have performed the largest mutational analyses in MUP metastatic nodes specimens and more comprehensive survival evaluation in respect to *BRAF*/*NRAS* status than has been published to date.^23^,^24^

Mutation distribution in the MUP study group was similar to those observed in other groups with melanoma of known primary site at stage III and IV and included 53 % of *BRAF* mutants (with the p.V600E as the most prevalent mutation) and 14 % of mutually exclusive *NRAS* mutants.^6^,^16^,^18^,^25^–^27^ No *KIT* mutations were found.

Based on our mutational data we have shown that a population of MUP patients have a similar distribution of BRAF/NRAS alterations to the known primary melanoma patients exposed to similar/identical environmental factors (such as UV exposure, but not exclusively), confirming data from other smaller published series.^16^,^18^,^23^,^24^,^28^ The incidence of mutations is consistent with those already observed for melanoma originating on skin without chronic sun damage, which is the most common type in Central and Western Europe.^29^ It implies common pathogenesis for tumor growth of MUP and other skin melanoma types.

There is no unambiguous theory on MUP development mechanism. Widely acceptable is the assumption of spontaneous, immune-induced, complete regression of the primary lesion preceding clinical metastases.^30^ In fact, melanoma is the most common tumor to undergo partial, severe regression, and such events are well documented in primary cutaneous melanoma with frequency of 3–8 %.^31^ Based on our knowledge of melanoma biology and clinical course, other explanations of MUP origins are also possible and include manifestation as a synchronous, unrecognized melanoma in multiple lesions or malignant transformation of benign nevus cells de novo in a lymph nodes.

The outcomes of our study are especially interesting when compared with reports conducted on different MUP patient populations, where other types of melanoma are dominant. Kong et al.^32^ reported *KIT* alternations in 13.7 % (including 7.8 % *KIT* mutations) in a large group of Chinese MUP patients. This is interesting, since acral-lentiginous type of melanoma with higher frequency of *KIT* alternations is the more frequent in Asian patients. However, it could also allude to a proportion of unrecognized mucosal melanomas.

Patients with melanoma of primary unknown site tend to have a prognosis and natural history of disease that is similar to, if not more favorable than, patients with the same staging characteristics, from a known primary cutaneous melanoma.^5^–^7^,^33^–^36^ In our group of MUP patients, survival rates are comparable to previously reported series (5-year OS rates ranged between 28.6 and 75.6 %) as well as series of stage III patients with macrometastases used for validation of AJCC staging system.^7^,^33^ It seems that our multicenter group of MUP patients with nodal involvement have outcomes not better than patients with detectable primary tumor according to AJCC database. It implies that they may derive from undetectable primary lesions in the past that gave nodal metastases with lead time bias. However, the results should be interpreted with caution, because alternative explanation of de novo development of the tumor within lymph node experiencing a different microenvironment from the start, resulting in aggressive tumor behavior, is also possible.

There is no agreement on prognostic features in MUP with nodal metastases; however, most commonly accepted are female gender, younger age at diagnosis, and smaller number of lymph nodes involvement.^4^–^7^ We have demonstrated that MUP stage III patients have the same important prognostic factor as known primary melanomas with clinically detected lymph node metastases (stage IIIB, C), e.g., nodal metastases burden expressed as number of metastatic nodes.^35^

The nodal metastases in MUP patients harboring *BRAF* mutation has a tendency to occur in younger age, which is consistent with observation that p.V600E *BRAF*-mutated melanomas more frequently affect younger individuals with lower cumulative UV exposure.^25^,^37^,^38^ OS was not dependent on mutational status (although a trend for poorer prognosis in *BRAF*/*NRAS* mutated cases was visible without reaching statistical significance, which may be related to insufficient number of patients); this has been seen previously in advanced melanomas.^11^,^18^ This study did demonstrate that the presence of *BRAF* and *NRAS* mutations had negative impact on DFS in this group of patients.^19^,^39^

Because the manifestation of melanoma as MUP does not alter *BRAF* or *NRAS* mutation frequency compared with melanomas with known primary site, some other genetic/immunological mechanism enhancing regression must be involved.^28^ Based on previously published data and our clinical data on MUP patients, supported by mutational status of known genes involved in development of melanoma as well as potential targets of new therapies, we conclude that MUP has similar molecular pattern in terms of *BRAF*/*NRAS* alterations and clinical behavior to melanoma of known primary site of the same stage (mainly developed in NCSD) and therefore should be managed and treated according to the same guidelines. It has important implications for potential adjuvant therapy as well as treatment of metastatic disease in an era of personalized medicine and strongly suggests that MUP with nodal involvement should be included for clinical trials with targeted therapy at stage III disease. *BRAF* and *NRAS* gene mutational status does not affect patient outcome significantly, although patients harboring mutations in those genes have less favorable DFS.
